# Pea Efficiency of Post-drought Recovery Relies on the Strategy to Fine-Tune Nitrogen Nutrition

**DOI:** 10.3389/fpls.2020.00204

**Published:** 2020-02-27

**Authors:** Mégane Couchoud, Christophe Salon, Sylvie Girodet, Christian Jeudy, Vanessa Vernoud, Marion Prudent

**Affiliations:** Agroécologie, AgroSup Dijon, INRAE, Université de Bourgogne, Université Bourgogne Franche-Comté, Dijon, France

**Keywords:** agroecology, water deficit, grain legumes, *Pisum sativum*, resilience, roots, symbiotic nitrogen fixation, yield stability

## Abstract

As drought is increasingly frequent in the context of climate change it is a major constraint for crop growth and yield. The ability of plants to maintain their yield in response to drought depends not only on their ability to tolerate drought, but also on their capacity to subsequently recover. Post-stress recovery can indeed be decisive for drought resilience and yield stability. Pea (*Pisum sativum*), as a legume, has the capacity to fix atmospheric nitrogen through its symbiotic interaction with soil bacteria within root nodules. Biological nitrogen fixation is highly sensitive to drought which can impact plant nitrogen nutrition and growth. Our study aimed at dynamically evaluating whether the control of plant N status after drought could affect nodulated pea plant’s ability to recover. Two pea genotypes, Puget and Kayanne, displaying different drought resilience abilities were compared for their capacity to tolerate to, and to recover from, a 2-weeks water-deficit period applied before flowering. Physiological processes were studied in this time-series experiment using a conceptual structure–function analysis framework focusing on whole plant carbon, nitrogen, and water fluxes combined to two ^13^CO_2_ and ^15^N_2_ labeling experiments. While Puget showed a yield decrease compared to well-watered plants, Kayanne was able to maintain its yield. During the recovery period, genotype-dependent strategies were observed. The analysis of the synchronization of carbon, nitrogen, and water related traits dynamics during the recovery period and at the whole plant level, revealed that plant growth recovery was tightly linked to N nutrition. In Puget, the initiation of new nodules after water deficit was delayed compared to control plants, and additional nodules developed, while in Kayanne the formation of nodules was both rapidly and strictly re-adjusted to plant growth needs, allowing a full recovery. Our study suggested that a rapid re-launch of N acquisition, associated with a fine-tuning of nodule formation during the post-stress period is essential for efficient drought resilience in pea leading to yield stability.

## Introduction

Pea (*Pisum sativum* L.) produces seeds rich in proteins (about 25%, Reichert and MacKenzie, 1982), which can be used for both feed and food. Like other legumes, pea does not require nitrogen fertilizer for its growth, making it an economic and environmental friendly crop that can play an important role in sustainable agriculture (Graham, 2003; Jensen and Hauggaard-Nielsen, 2003). This particular feature is due to legumes’ unique ability to fix atmospheric dinitrogen (N_2_) thanks to a symbiosis with soil bacteria (type Rhizobia) inside specific root structures called nodules. The symbiotic nitrogen fixation (SNF) process relies on structural components (nodule number and size), and on functional characteristics (N_2_ fixation thanks to the nodule nitrogenase enzyme). Photosynthetic carbon arising from shoots sustains nodules formation, nodule maintenance and N_2_ fixation activity (Kouchi and Yoneyama, 1984) at the expense of both shoot growth and root growth and functioning (Salon et al., 2001; Voisin et al., 2003a, b). The nodulation process is thus strictly adjusted to plant growth and is controlled by both local control mechanisms and a systemic regulation process known as “autoregulation of nodulation” (Reid et al., 2011) which presumably depends on the nitrogen status of the plant (Ruffel et al., 2008; Jeudy et al., 2010). This results in the induction of synchronous and transient waves of nodulations in the root system.

In the current context of climate change, we are witnessing an increase in the heterogeneity of rainfall with alternating periods of heavy rainfall and drought (Bernstein et al., 2007; Dai, 2013), causing plant yield and seed protein content instability, and contributing as an example to the decrease in the cultivated area of pea in Europe (Cernay et al., 2015). One of the first responses observed under water deficit, which contributes to plant tolerance is stomatal closure mediated by abscisic acid (ABA), leading to a decrease in stomatal conductance (Chaves et al., 2003; Anjum et al., 2011). While stomatal closure reduces water loss, it can also decrease plant photosynthesis and thus reduce biomass accumulation (Chaves et al., 2003; Xu et al., 2010). Water deficit also modulates biomass partitioning among the different plant compartments of legumes: root growth is maintained or even increased at the expense of shoot growth (Xu et al., 2010; Kunert et al., 2016; Prudent et al., 2016) while nodule growth is reduced (Mahieu et al., 2009; Prudent et al., 2016). SNF is highly sensitive to environmental constraints (Salon et al., 2001) and particularly to water deficit (Zahran, 1999). The specific nitrogen fixation activity (sNFA) is greatly decreased by water deficit (Serraj et al., 1999; Streeter, 2003; Prudent et al., 2016) as a result of several factors including O_2_ partial pressure within nodules, N feedback regulation and carbon limitation (Larrainzar et al., 2009). Thus, increasing occurrences of drought will exacerbate the negative impact of water deficit on SNF (Vadez et al., 2012), which is detrimental to legumes nitrogen nutrition under water deficit conditions, and thus to yield stability.

When a plant faces a stress, its tolerance to the stress and its recovery after the stress constitute two different processes which contribute to its resilience. Hodgson et al. (2015) define resilience as the outcome of tolerance and recovery, the tolerance being “the impact of exogenous disturbance” and the recovery being all “endogenous processes that pull the disturbance system back toward an equilibrium.” Post-stress recovery corresponds to an important part of resilience of ecosystems (Lake, 2013) which has not been yet extensively studied as compared to plant tolerance. Plant performance during a stress is not necessarily correlated to its ability to recover post-stress (Striker, 2012). This highlights the importance of taking into consideration the mechanisms underlying plant recovery to improve crop stress resilience.

Plant’s ability to recover from a stress is essential for its survival and yield establishment, especially after a drought period (Chaves et al., 2009). In maize it has been suggested that drought recovery could play a more important role in plant resilience than drought resistance (Chen et al., 2016). A complete recovery after water deficit has been observed in different species such as maize (Sun et al., 2016), *Medicago* (Yousfi et al., 2015), chickpea (Yaqoob et al., 2013), and pea (Prudent et al., 2016). Although post-drought recovery is being increasingly studied, the available studies have generally focused only on a single harvest after the re-watering period, which lasted from a few days to weeks (da Silveira et al., 2001; Naya et al., 2007; Larrainzar et al., 2009; Nasr Esfahani et al., 2014; Prudent et al., 2016, 2020). Such time points of observation and lack of dynamics are not sufficient to fully assess the mechanisms underlying plant recovery as it presumably comprises a diversity of structural and functional responses, leading to either partial, complete, or over compensatory recoveries. The recovery of a process after a disturbance can be explained by various parameters such as the latency time to initiate the recovery, the rate of the process’s response, the time taken to reach a stable state, and the gap (Δ) between the value of the process in a disturbed situation and in the control condition (Hodgson et al., 2015). As such, it is essential to consider the dynamic dimension of the recovery. Moreover, a time-series study of post-drought recovery in the model legume *Medicago truncatula* suggested that the nutritional status of the plant could also shape its ability to recover (Lyon et al., 2016). Lastly, the importance of the source of legume N nutrition (nitrate-fed versus SNF) in recovery dynamics was also highlighted as nodulated plants recovered faster than nitrate-fed plants after drought (Staudinger et al., 2016). We thus decided to explore whether the control of plant N status after drought could affect plant ability to recover, when plant N nutrition relies only on SNF. We hypothesized that a quick and strict adjustment of the number of N_2_-fixing structures (i.e., nodules) to plant growth needs is a key trait for an efficient post-drought recovery.

To that aim, we characterized N nutrition during drought and subsequent recovery in two pea genotypes displaying contrasted drought resiliences. In order to assess mechanisms involved in both tolerance and recovery mechanisms, pea plant responses during water deficit and subsequent re-watering were dynamically analyzed in two independent experiments, the second involving two successive plant labeling experiments. Because N nutrition is tightly linked at the whole plant level to carbon and water nutrition (especially under drought), and specifically for nodules (Liu et al., 2018), physiological processes were studied using a conceptual structure–function analysis framework focusing mainly on plant C, N, and water fluxes.

## Materials and Methods

### Plant Growth Conditions

Two genotypes of *Pisum sativum* L. were used: Kayanne, an *afila* spring pea cultivar (obtained from KWS Momont, Mons-en-Pévèle, France) and Puget, a leaflet garden pea cultivar (obtained from Graines-LORAS, La Tour-de-Salvagny, France). Both cultivars have a semi-determinate growth habit. Seeds were calibrated and pre-germinated at 21°C in the dark during 4 days. After being transplanted in 2-L pots filled with a mixture of perlite:sand (3:1, v:v) seedlings were inoculated with 1 ml of *Rhizobium leguminosarum* bv. *viciae*, strain P221 (MIAE01212, 10^8^ bacteria.ml^–1^). Plants were transferred in a greenhouse of the Plant Phenotyping Platform for Plant and Microorganism Interactions (4PMI) at INRAE in Dijon (France) 1 week after sowing. Mean day/night temperatures were 20/16°C and the photoperiod was 16 h. Artificial light (PAR of 280 μmol.m^–2^.s^–1^) was supplied by sodium lamps (MACS 400W; Mazda, Dijon, France) to complement natural light during the photoperiod.

During the first 2 weeks, plants were automatically watered four times a day with a N-free nutritive solution to reach 100% of substrate water-holding capacity. Substrate water-holding capacity was gravimetrically estimated before each watering (for more details, see Supplementary Figure S1). Plants of each genotype were then split in two groups: half of the plants (Well-Watered plants, hereafter referred as WW) was maintained until physiological maturity under optimal water conditions corresponding to 100% of substrate water-holding capacity. The other half of plants (Water-Deficit plants, hereafter referred as WD plants) was subjected to a water-deficit period of 2 weeks by withholding water until pots reached 40% of substrate water-holding capacity (Supplementary Figure S1), corresponding to a predawn plant water potential of −0.9 MPa. WD plants were then re-watered to reach 100% of substrate water-holding capacity until physiological maturity (Supplementary Figure S1).

For the labeling experiment, plant growth conditions were similar, except than individual air-tight PVC 2-L pots were used to allow shoot-root atmosphere separation. Mean day/night temperatures were set at 20/17.5°C and artificial light (PAR of 250 μmol.m^–2^.s^–1^) was supplied by sodium lamps (MACS 400W; Mazda, Dijon, France) to complement natural light during the photoperiod.

### Experimental Design and Sampling

**FIGURE 1:**
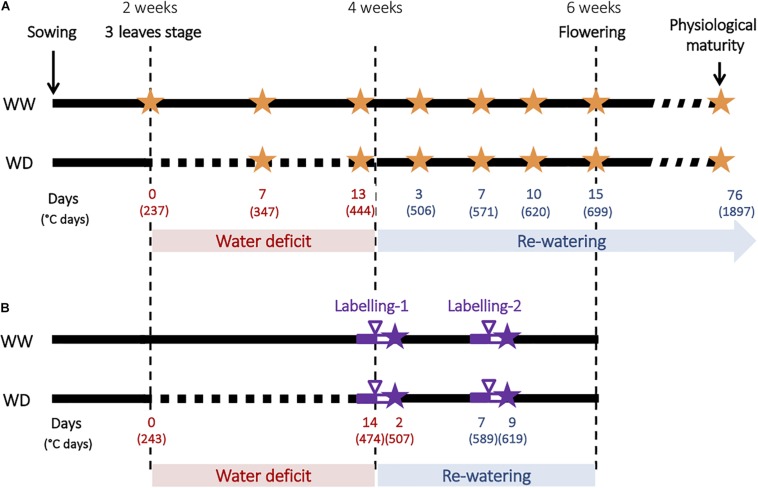
Experimental design used to characterize plants responses to water deficit and subsequent re-watering. **(A)** Description of the kinetics experiment. Water deficit was imposed during the vegetative stage for 2 weeks and followed by a re-watering period. WW corresponds to well-watered plants and WD corresponds to plant subjected to water deficit, orange stars indicate harvests. Time is expressed in days and degree days. **(B)** Description of the ^13^CO_2_ and ^15^N_2_ labeling experiments. Full purple line indicates acclimation period, purple arrowhead indicates labeling, empty purple line indicates chase period and purple stars indicate harvests.

For the first kinetics experiment (Figure 1A), six plants per genotype and per water treatment were harvested at seven different times corresponding to the beginning, the middle (1 week) and the end (2 weeks) of the water-deficit period, and to 3, 7, 10, and 15 days of re-watering (Figure 1A). For each harvest, six plants per condition were sampled. Shoots and roots were separated. Leaves and stem from shoots were separated during the vegetative period and additionally pods and seeds were harvested during the reproductive stage. The nodulated root system was gently washed, and nodules were manually removed from the root system, as soon as they were visible at the naked eye, and counted. At physiological maturity, 10–12 plants per condition were harvested.

For the second experiment involving labeling, carbon and nitrogen were labeled with ^13^CO_2_ and ^15^N_2_ respectively at two periods during plant growth, the first at the end of the water-deficit period and the second after 7 days of re-watering (Figure 1). For each labeling experiment, six pots of each condition and genotype were transferred 2 days before labeling from the greenhouse (see above) to a transparent air-tight labeling chamber made of Plexiglas for acclimation. Shoot and root atmospheres were controlled using Dasylab software (SM2i, Villiers-Saint-Frédéric, France), and temperature and humidity were regulated by using an air conditioning unit (Voilot, Dijon, France). Mean day/night temperatures were 21/17.5°C and the photoperiod was 10 h supplied by lamp (Osram, Dulux L, 55W 954) leading to an average PAR of 535 μmol.m^–2^.s^–1^.

The day of labeling, PVC pots were closed and air tightness was ensured using physiological molding material (Qubitac, Qubit System, Inc., Kingston, ON, Canada) and silicone rubber (RTV 65RTV3428-1, Zundel & Kohler, France). Shoots were exposed to a ^13^CO_2_-enriched atmosphere for 10 h, with an air CO_2_ concentration of 380 ppm and a ^13^CO_2_ enrichment of 10 Atom%. During this period, air CO_2_ concentration and ^13^CO_2_ enrichment were continuously measured with an infrared gas analyzer (IRGA S710, Sick Maihak AG, Hamburg, Germany) and maintained by automatic injection of a mixture of ^13^CO_2_ and ^12^CO_2_. Nodulated roots were simultaneously exposed to a ^15^N_2_-enriched atmosphere by direct injection of ^15^N_2_ (5% ^15^N/^14^N) into air-tight pots for 24 h (simultaneously to the 10 h of ^13^CO_2_ shoot exposure and the following night). The amount of ^15^N_2_ to be injected was determined at the outset by measuring the mean air volume in the container. To obtain an accurate measurement of ^15^N/^14^N enrichment in each pot, aliquots were sampled 30 min, 10 and 24 h after ^15^N_2_ injection with five replicates per sampling.

To study C and N isotope partitioning among plant parts, labeling was followed by a 2 days chase period where plants were exposed to an atmosphere with natural C and N enrichment and then harvested similarly to the kinetics experiment.

### Measurement and Calculation

Our framework of analysis was structure–function based and enriched the version of Moreau et al. (2012) by adding nodule N acquisition and water fluxes (Supplementary Figure S2). It considered “structural variables,” characterizing plant growth including plant, shoot, nodulated root, nodule and root biomass, leaf area, nodule number, plant nitrogen amount, and concentration and evapotranspiration. These variables are linked together by “functional variables,” characterizing plant capacity to uptake and use resources (carbon, nitrogen, and water): nitrogen use efficiency (NUE), radiation use efficiency (RUE), sNFA, water use efficiency (WUE), specific root water uptake (sRWU).

Evapotranspiration (gH_2_0) was gravimetrically determined, based on the daily water loss from each pot, by calculating the difference between pot weight before and after each watering. Stomatal conductance (mmol water.m^–2^.s^–1^) was measured with a diffusion porometer (AP4; Delta T device, Cambridge, United Kingdom) on a stipule of the last fully expanded leaf.

For each harvest, all plant compartments (root, nodule, leaf, stem and pod and seed) were dried for 48h in an oven at 80°C. Biomass (BM) of each dried part was measured and then dried tissues were ground into a fine powder and analyzed with an elemental analyzer (Thermo Electron NC2500, Courtaboeuf, France) in order to estimate C and N concentration in each tissue.

For each plant, shoot/nodulated root ratio corresponding to the ratio of above-ground biomass (leaves and stem) over total below-ground dry biomass (roots and nodules) were calculated. The nodule/nodulated root ratio corresponding to nodule biomass divided by total below-ground biomass, was also calculated.

For leaf area measurement, leaves were spread out and scanned on a scanner (EPSON GT20000, Model J151A, Japan), and leaf area was measured using the WinRhizo software (Regent Instruments Canada, Inc., 2012b version).

The Nitrogen Nutrition Index (NNI) was calculated as the ratio between shoot N concentration and the critical N concentration (Nc), following: Nc = 4.756 BM^–0.088^ (Voisin et al., 2015).

Radiation use efficiency between date t_1_ and t_2_ was calculated as the ratio of the plant biomass (BM) accumulated during this period over the integrated leaf area between t_1_ and t_2_ and is expressed in g.cm^–2^:

$$\text{RUE}=\frac{B\,M_{t\,2}-B\,M_{t\,1}}{\int_{1}^{2}\mathrm{leaf}\,\mathrm{area}.\text{dt}}$$

Nitrogen use efficiency between date t_1_ and t_2_ was calculated as the ratio of the plant biomass (BM) over the plant nitrogen amount (QN) accumulated during this period and is expressed in g.gN^–1^:

$$\text{NUE}=\frac{B\,M_{t\,2}-B\,M_{t\,1}}{Q\,N_{t\,2}-Q\,N_{t\,1}}$$

Water use efficiency between date t_1_ and t_2_ was calculated as the ratio of the plant biomass (BM) accumulated over the amount of water evapotranspired by the plant during this period (QH_2_O) and is expressed in g. gH_2_O^–1^:

$$\text{WUE}=\frac{B\,M_{t\,2}-B\,M_{t\,1}}{Q\,H\,2\,O_{t\,2}-Q\,H\,2\,O_{t\,1}}$$

The specific Root Water Uptake between date t_1_ and t_2_ was calculated as the ratio of the amount of water evapotranspired by the plant (QH_2_O) over the integrated root dry weight (BM_root_) between t_1_ et t_2_ and is expressed in gH_2_O[gBM_root_ day^–1^] ^–1^:

$$\text{sRWU}=\frac{Q\,H\,2\,O_{1\to 2}}{\int_{1}^{2}{\text{BM}}_{r\,o\,o\,t}.\text{dt}}$$

### ^13^C and ^15^N Content and Enrichment Determination

For each harvest, all plant compartments (root, nodule, leaf, stem) were dried for 48 h in an oven at 80°C. The biomass of each part was measured and then dried tissues were ground into a fine powder. Aliquots were analyzed for their C and N concentrations with an elemental analyzer (EA, VarioMicroCube, Elementar) and their ^13^C and ^15^N enrichment determined with an online isotope ratio mass spectrometer (irm-EA/MS, Isoprime/Elementar).

### Isotopic Calculations for the Labeling Experiments

Calculations were done according to the isotopic dilution principle. The percentage of C or N allocated to the different compartments during the labeling period was calculated as the ratio of C or N incorporated in the compartment i (QCl_i_ or QNl_i_) during the labeling experiment over the total amount of C or N incorporated in plant (QCl_plant_ or QNl_plant_) during this period:

$$\text{C allocation to compartment i}=\left(\frac{{\text{QCl}}_{\text{i}}}{{\text{QCl}}_{\text{plant}}}\right)\times 100$$

$$\text{N allocation to compartment i}=\left(\frac{{\text{QNl}}_{\text{i}}}{{\text{QNl}}_{\text{plant}}}\right)\times 100$$

where QCl and QNl can be calculated as:

$$\text{QCl}=\text{BM}\times \text{(\%C/100)}\times \left(\frac{\left({\text{AC}}_{\text{organ}}-{\text{AC}}_{\text{control}}\right)}{\left({\text{AC}}_{\text{source}}-{\text{AC}}_{\text{control}}\right)}\right)$$

$$\text{QNl}=B\,M\times \text{(\%N/100)}\times \left(\frac{\left({\text{AN}}_{\text{organ}}-{\text{AN}}_{\text{control}}\right)}{\left({\text{AN}}_{\text{source}}-{\text{AN}}_{\text{control}}\right)}\right)$$

where AC_organ_ and AN_organ_ are the ^13^C and ^15^N abundances, respectively, of the plant organ of the labeled plant or non-labeled control plants; AC_source_ and AN_source_ are the ^13^C and ^15^N enrichment, respectively, of the labeled shoot and root atmosphere; BM is biomass and %C and %N are the percentages of C and N in biomass (w/w), respectively.

sNFA (gN.gBM_nodule_^–1^.day^–1^) was calculated as the ratio of total amount of N incorporated in plant during the labeling experiment in plant (QNl_plant_) over nodule biomass (BM_nodule_):

$$\text{sNFA}=\frac{{\text{QNl}}_{\text{plant}}}{{\text{BM}}_{\text{nodule}}}$$

Photosynthesis during the labeling day (gC.gBM_shoot_^–1^.day^–1^) was calculated as the ratio of total amount of C incorporated in plant (QCl_plant_) during the labeling experiment over shoot biomass (BM_shoot_):

$$\text{Photosynthesis}=\frac{{\text{QCl}}_{\text{plant}}}{{\text{BM}}_{\text{shoot}}}$$

The labeling technique used for measuring net carbon photosynthetic input into plant tissue is much more precise than relying on a “budget method” (i.e., weighting plants and measuring the increment of stable ^12^C content). The tracer which here is labeled ^13^CO_2_ is introduced at a known enrichment far above the natural one (around 1,1%) and precise IRMS measurements allow assessing the contribution of unlabeled ^12^C previously incorporated in plants vs. the ^13^C provided to plants during the labeling period.” Advantages of isotopic labeling and fluxomics have been reviewed in Salon et al. (2017).

### Statistical Analyses

All statistical analyses were performed with R software, version 3.5.3. An analysis of variance was performed using “aov” function and significant differences among conditions were determined by SNK test (“SNK.test” function from “agricolae” Package, version 1.3.1). Results from the analysis of variance are shown in Supplementary Table S1. For efficiency and activity variables, a bootstrap sampling method was used.

Student’s *t*-tests were performed for each genotype to test for water treatment effect (“t.test” function). Only differences at the 0.05 probability level were considered.

## Results

### Kayanne and Puget Which Have Contrasted Plant Architecture Display Different Resilience Abilities Toward Water Deficit

Two pea genotypes were studied: Puget is a leaflet genotype while Kayanne is a genotype with the *afila* allele which transforms leaflets into tendrils (Supplementary Figure S3). This leads to a lower leaf area in Kayanne than in Puget. However, the shoot-to-root ratio of Puget is lower than that of Kayanne, due to the greater development of its root system (Supplementary Table S1).

**FIGURE 2:**
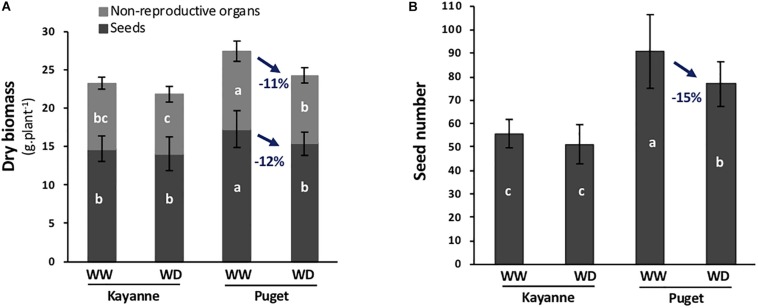
Plant yield and yield components at physiological maturity are affected by water deficit applied during the vegetative stage followed by a period of re-watering. **(A)** Total plant biomass was divided into seed biomass (dark gray) and non-reproductive organ biomass (light gray). **(B)** Number of seeds per plant. Values are means, bars represent standard deviations (*n* = 12). Different letters indicate statistically significant differences between treatments (ANOVA followed by SNK test, *p* < 0.05).

Two week-old Kayanne and Puget plantlets were subjected to a moderate and progressive water deficit after which plants were optimally re-watered allowing a period of recovery until harvest (Figure 1A). The effect of water deficit on both genotypes was evaluated at physiological maturity by measuring plant yield and yield components. Water deficit decreased Puget’s plant and seed biomasses, while Kayanne was not significantly affected (Figure 2A). The lower yield in Puget resulted from less seeds (−15%, Figure 2B) rather than from a decrease in individual seed weight. This indicates that Kayanne was more resilient toward a moderate 2-week water deficit applied during the vegetative phase than Puget.

Stress tolerance and recovery capacities of both genotypes were compared during the water deficit period (3 sampling dates), and during the first 2 weeks of the re-watering phase (4 sampling dates, Figure 1A). Two types of plant variables were evaluated during this kinetics experiment: “structure variables” including plant biomass, plant leaf area and plant N amount, and “functional variables” characterizing the plants’ capacity to uptake and use carbon, nitrogen and water resources, such as RUE, NUE, and WUE. These variables were linked together in a conceptual structure–function framework of plant functioning that was the basis of our analysis (Supplementary Figure S2). Each variable was measured or calculated for each sampling date, condition and genotype and will be presented in the manuscript as a percentage relative to the control plants. Primary data are available in Supplementary Table S1.

### Plant Growth Was Similarly Impacted During Water Deficit Whatever the Genotype While Plant Growth Recovery Initiation Was Genotype Dependent

**FIGURE 3:**
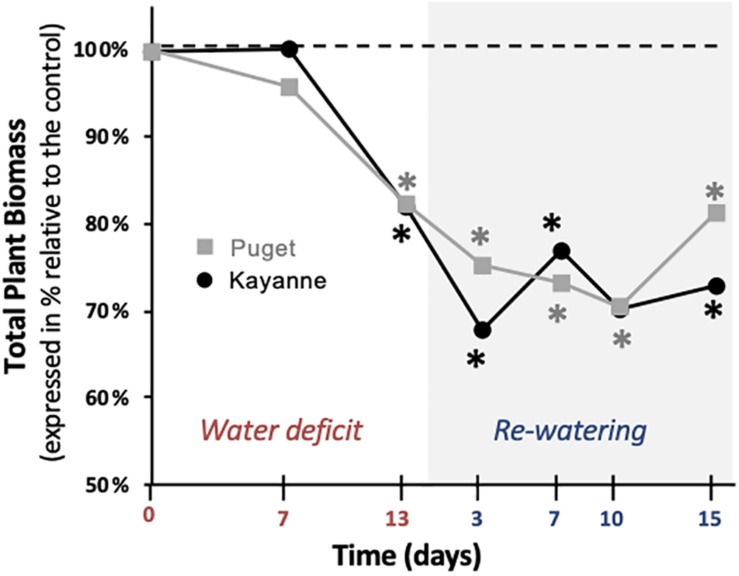
Plant growth during water deficit and subsequent re-watering. Total plant biomass was measured during the water deficit period (0, 7, 13 days) and after 3, 7, 10, and 15 days of re-watering. For each genotype, data are presented as a percentage relative to the control plants. Asterisks indicate Student’s *t*-test significant differences between control and water deficit plants for a given genotype (black asterisk for Kayanne, gray asterisk for Puget; *p* < 0.05, *n* = 6). Primary data are available in Supplementary Table S1.

Total plant biomass is at the center of our conceptual framework (Supplementary Figure S2). Whatever the genotype, total plant biomass of WD plants, was reduced at the end of the stress period by around 18% as compared to WW plants (Figure 3). During re-watering, the sequence of Puget and Kayanne recoveries differed. The initiation of recovery, illustrated by an increase of the total plant biomass when expressed relative to the control, occurred between 3 and 7 days in Kayanne and between 10 and 15 days for Puget. After 2 weeks of re-watering, plant biomass did not fully recover, as compared to WW plants, for either genotype (Figure 3).

### Water Deficit Affects Water Fluxes in Both Genotypes

**FIGURE 4:**
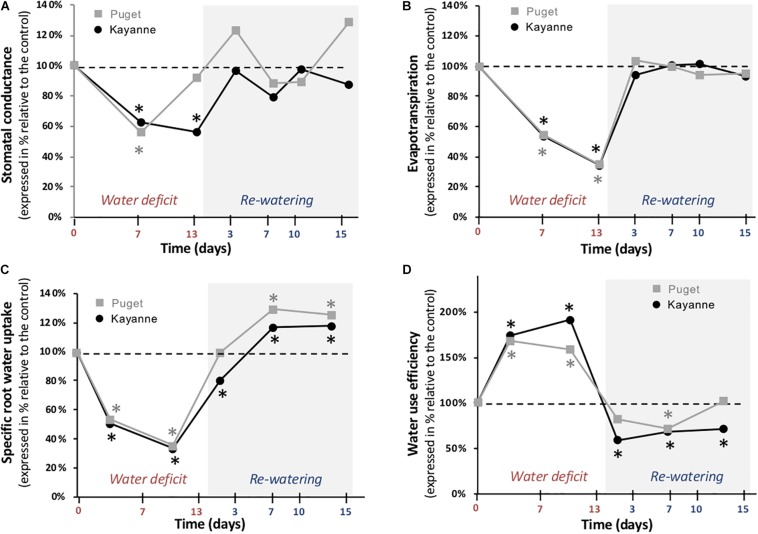
Water fluxes during water deficit and subsequent re-watering. **(A)** Stomatal conductance and **(B)** evapotranspiration were measured during the water deficit period (0, 7, 13 days) and after 3, 7, 10, and 15 days of re-watering. **(C)** Specific root water uptake and **(D)** water use efficiency were calculated between two successive harvests. Genotype Kayanne is in black and Puget in gray. For each genotype, data are presented as a percentage relative to the control plants. Asterisks indicate Student’s *t*-test significant differences between control and water deficit plants for a given genotype (black asterisk for Kayanne, gray asterisk for Puget; *p* < 0.05, *n* = 6). Primary data are available in Supplementary Table S1.

Biomass accumulation and water management are intimately linked and rely on plant ability to both uptake and use water (Supplementary Figure S2). The effects of water deficit and subsequent re-watering on plant water fluxes were thus evaluated by measuring stomatal conductance, evapotranspiration, sRWU, and WUE (Figure 4). After 7 days of water deficit, stomatal conductance was reduced by 38 and 44% in Kayanne and Puget, respectively (Figure 4A). By the end of the water deficit period, stomatal conductance of WD Kayanne decreased by 56% compared to the WW plants, while stomatal conductance of WD Puget returned to values similar to the control. This complete stomatal conductance recovery occurred 3 days post-stress in Kayanne (Figure 4A). In terms of evapotranspiration, both genotypes behaved similarly, with a great reduction during water deficit (−65%) and a rapid and complete recovery observable at 3 days of re-watering (Figure 4B). Synchronized with the decrease in evapotranspiration during the WD period, a major reduction of about 60% of the sRWU for both genotypes was observed. Following WD release, sRWU rapidly increased and even exceeded that of WW plants and this was maintained throughout the 2-week re-watering period (Figure 4C). WUE increased in response to water deficit by 75 and 68% for Kayanne and Puget respectively. During the re-watering period, WUE of WD plants decreased below that of control plants. After 2 weeks of re-watering, while WD plants of Puget displayed WUE similar to that of control plants, the WUE of WD plants of Kayanne was still 28% lower than that of WW plants (Figure 4D). Altogether these data show (i) a complete recovery for all the functional variables related to water fluxes for both genotypes, except for the WUE of the WD Kayanne plants and (ii) a delayed recovery for water uptake and use in Kayanne compared to Puget.

### Shoot Functional but Not Structural Components Recovered Fully After 2 Weeks of Re-watering

**FIGURE 5:**
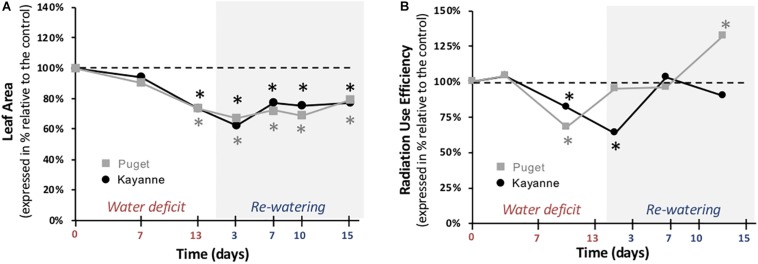
Leaf area and radiation use efficiency during water deficit and subsequent re-watering. **(A)** Leaf area was measured during the water deficit period (0, 7, 13 days) and after 3, 7, 10, and 15 days of re-watering. **(B)** Radiation use efficiency was calculated between two successive harvests. Kayanne is in black and Puget in gray. For each genotype, data are presented as a percentage relative to the control plants. Asterisks indicate Student’s *t*-test significant differences between control and water deficit plants for a given genotype (black asterisk for Kayanne, gray asterisk for Puget; *p* < 0.05, *n* = 6). Primary data are available in Supplementary Table S1.

Accumulation of biomass depends on structural components including leaf area which supports several functions such as photosynthesis and conversion of light radiation to biomass, called RUE (Supplementary Figure S2). In response to water deficit, leaf areas of both genotypes were similarly decreased as compared to WW plants at the end of the water-deficit period (by about 26%) and after 2 weeks of re-watering (by 23 and 21% for Kayanne and Puget, respectively) (Figure 5A). RUE was decreased by 20 and 31% for Kayanne and Puget respectively during water deficit (Figure 5B). During the re-watering period, RUE quickly and completely recovered after 1 and 7 days for Puget and Kayanne, respectively. However, while RUE was maintained similar to WW values in Kayanne, RUE of Puget overcompensated and was 33% higher than the control plants after 2 weeks of re-watering (Figure 5B). These results underline that shoot structural and functional components were negatively impacted by water deficit but that only functional components fully recovered after 2 weeks of re-watering. Results also demonstrate that Kayanne’s shoot functional recovery occurred later than for Puget.

### Water Deficit and Subsequent Re-watering Triggered Changes in Biomass Allocation Toward the Nodule Compartment in a Genotype-Dependent Manner

**FIGURE 6:**
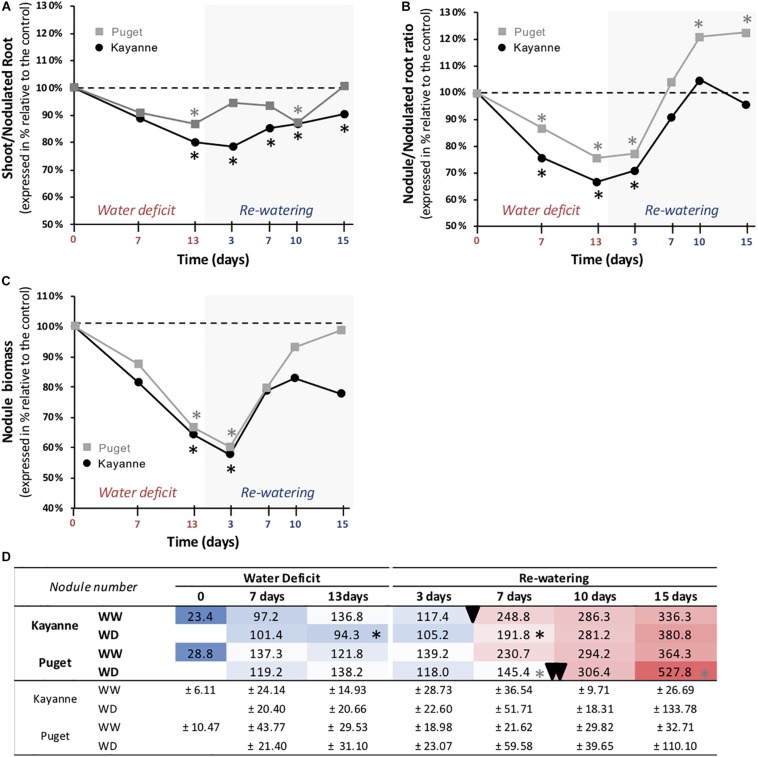
Biomass partitioning during water deficit and subsequent re-watering. **(A)** Shoot to root ratio, **(B)** nodule to nodulated root ratio, and **(C)** nodule biomass were calculated and measured during the water deficit period (0, 7, 13 days) and after 3, 7, 10, and 15 days of re-watering. Genotype Kayanne is in black and Puget in gray. For each genotype, data are presented as a percentage relative to the control plants. **(D)** Evolution of the nodule number during water deficit and re-watering. A delay in the establishment of the second wave of nodulation is shown for Puget WD plants during the re-watering period (double arrowhead) compared to Puget WW and Kayanne WW and WD plants (arrowhead). WW, well-watered; WD, water deficit. Data are means ± SD (*n* = 6). Asterisks indicate Student’s *t*-test significant differences between control and water deficit plants for a given genotype (black asterisk for Kayanne, gray asterisk for Puget; *p* < 0.05, *n* = 6). Primary data are available in Supplementary Table S1.

We next determined how water deficit and subsequent re-watering affected biomass partitioning between above- and below-ground organs. The evolution of the shoot/nodulated root biomass ratio is presented in Figure 6A. At the end of the water-deficit period, this ratio was lower for Kayanne plants subjected to WD by 20% when compared to the WW plants. This trend was maintained during the re-watering period and could be explained by a maintenance of root growth together with a decrease of shoot growth (see Supplementary Table S1 for individual values of roots and shoot biomasses). On the contrary, the shoot/nodulated root ratio of Puget recovered quickly during the re-watering period (Figure 6A).

Within the nodulated root system, biomass allocation in nodules decreased during the water deficit period as revealed by the reduction of the nodule/nodulated root ratio for both Kayanne (−33% after 13 days of stress) and Puget (−24%) (Figure 6B). This mainly resulted from decreased growth of pre-existing nodules (Figure 6C), as the total number of nodules was not (Puget) or only slightly (Kayanne) affected (Figure 6D). After water deficit release, the nodule/nodulated root ratio of the rehydrated plants progressively re-increased for both genotypes reaching the control level after 1 week of re-watering (Figure 6B). Interestingly, while in Kayanne this ratio was maintained similar to that of WW plants during the second week of re-watering, it continued to increase in Puget, reaching a 23% higher value in WD plants compared to WW plants (Figure 6B). This overcompensation resulted from an increased nodule number: after 15 days of re-watering, rehydrated Puget roots developed about 45% more nodules than control plants (Figure 6D).

A marked increase in nodule number was observed between 3 and 7 days of re-watering and corresponded to the initiation of a second wave of nodulation for the control plants of both genotypes, and for the WD plants of Kayanne (Figure 6D). Interestingly in WD Puget plants, this increase occurred between 7 and 10 days of re-watering.

### Plant Nitrogen Status Was Negatively Impacted by Water Deficit Then Completely Recovered, but With a Different Timing Depending on the Genotype

**FIGURE 7:**
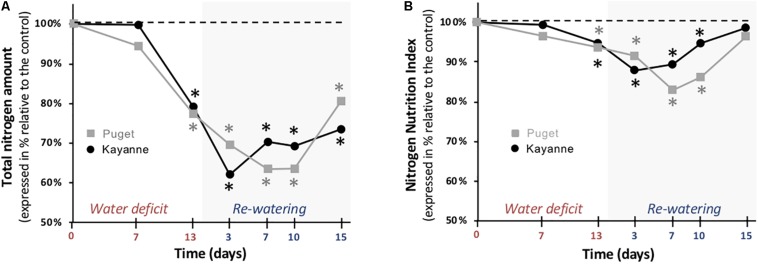
Plant nitrogen status during water deficit and subsequent re-watering. **(A)** Total nitrogen amount and **(B)** Nitrogen Nutrition Index (NNI) were determined during the water deficit period (0, 7, 13 days) and after 3, 7, and 15 days of re-watering. Genotype Kayanne is in black and Puget in gray. For each genotype, data are presented as a percentage relative to the control plants. Asterisks indicate Student’s *t*-test significant differences between control and water deficit plants for a given genotype (black asterisk for Kayanne, gray asterisk for Puget; *p* < 0.05, *n* = 6). Primary data are available in Supplementary Table S1.

Having shown that biomass allocation toward the nodule compartment was differently affected in Puget and Kayanne, particularly during post-stress recovery, we next examined whether this had an impact on plant nitrogen status. In both genotypes, the total nitrogen amount accumulated in plants was significantly impacted by water deficit (−23%), decreased to the same extent during the re-watering period (−38%), and partially recovered at the end of the 2-week re-watering period (Figure 7A). However, a difference in the timing of recovery was observed between genotypes: while it started after 3 days of re-watering in Kayanne, 10 days were required to initiate the recovery in Puget (Figure 7A). This difference in kinetics between the two genotypes was also observed for the plant nitrogen concentration (Supplementary Figure S4A), and for the plant N status, which was estimated by calculating the NNI (Figure 7B). In contrast to the total N amount, NNI fully recovered at the end of the 2-week re-watering period in both genotypes.

Nitrogen use efficiency (NUE) was calculated in order to evaluate plant’s ability to use accumulated N for biomass production (Supplementary Figure S4B). NUE was maintained for Puget and Kayanne during water deficit, but increased during the first days of the re-watering period up to 44% for Kayanne and 20% for Puget. Then NUE decreased to finish lower than that of control plants in both genotypes, but it occurred after 15 days of re-watering for Kayanne and 13 days for Puget (Supplementary Figure S4B).

### Combined ^13^CO_2_ and ^15^N_2_ Labeling Experiment Highlighted Different C and N Uptake Activities and Allocations Depending on the Genotype

Labeling experiments were performed in order to accurately assess how C and N uptake, and their allocation in the various plant organs were impacted during the water deficit period and after re-watering. The use of ^13^CO_2_ allowed to measure precisely C acquired by the plant through photosynthesis and its partitioning in the plant organs. Labeling plants with ^15^N_2_ provided us with a precise measurement of daily SNF activity for each of the labeling experiment. The first labeling was performed at the end of the water deficit period and the second after 1 week of re-watering (Figure 1B).

Water deficit decreased plant photosynthesis by 23 and 8% for Kayanne and Puget, respectively, but there was a complete recovery for both genotypes after 1 week of re-watering (Table 1). The sNFA was not significantly impacted at the end of the water deficit, neither for Puget nor for Kayanne (Table 1). After 1 week of re-watering, sNFA was 23% lower in WD plants than in WW plants only for Puget (Table 1).

**TABLE 1:** Photosynthesis and symbiotic nitrogen fixation activity (sNFA) after 2 weeks of water deficit and 1 week of re-watering.

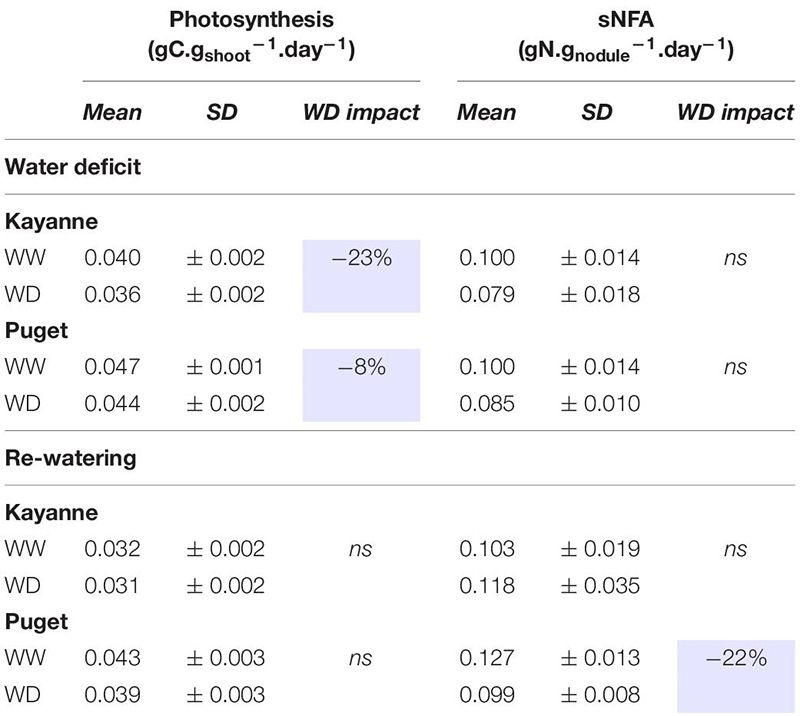

WW, well-watered plants; WD, plants subjected to water deficit. Values are means and SD standard deviations (*n* = 6). When differences between control and water deficit plants for a given genotype were significant (Student’s *t*-test; *p* < 0.05), the relative WD impact was calculated as (WD-WW) * 100/WW. *ns*, not significant.

The C and N labeling experiment allowed to compare C and N allocations to the different plant organs (shoot, root, and nodules) between the two genotypes (Table 2). In Kayanne only, water deficit increased C and N allocations to roots by 43 and 83%, respectively. However, C allocation to nodules was decreased for both genotypes, by 34% for Kayanne and 18% for Puget. After 1 week of re-watering, C and N allocations to roots and nodules of rehydrated Kayanne plants were similar to those of the control plants. For Puget, N allocations to roots was significantly reduced as compared (18%) to the control plants without any changes of C allocations. Regarding the nodule compartment, C and N allocations of water deficit plants reverted to values similar to those of control plants for Kayanne, but became higher than that of control plants (61 and 53% higher respectively) for Puget. This was in agreement with the evolution of the shoot/nodulated root ratio and the nodule/nodulated root ratio measured during the kinetics experiment (Figure 6).

**TABLE 2:** Carbon and nitrogen allocations after 2 weeks of water deficit and 1 week of re-watering.

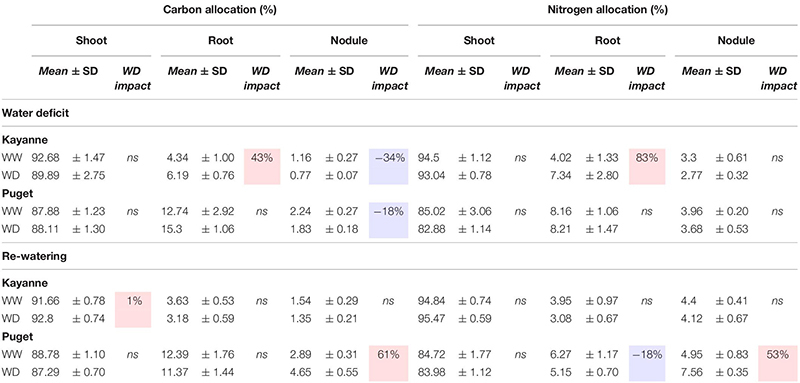

WW, well-watered plants; WD, plants subjected to water deficit. Values are means and SD standard deviations (*n* = 6). When differences between control and water deficit plants for a given genotype were significant (Student’s *t*-test; *p* < 0.05), the relative WD impact was calculated as (WD-WW) * 100/WW. *ns*, not significant.

In conclusion water deficit and subsequent re-watering caused changes in carbon and nitrogen allocations to the nodulated root system in a genotype-dependent manner.

## Discussion

Our study aimed at evaluating whether plant N nutrition could affect nodulated pea plant’s ability to recover after a drought period. Two pea genotypes displaying contrasted resilience abilities after a 2-week water deficit were studied: one (Kayanne) was able to maintain yield to the level of well-watered plants, while the other (Puget) was less resilient, showing a 12% decrease of yield in WD conditions (Figure 2). The major difference between the two genotypes under optimal watering conditions was that Kayanne allocated less biomass to underground organs (roots and nodules) than Puget (Supplementary Table S1), suggesting that different mechanisms in resource allocation and use could be established during drought tolerance and post-drought recovery.

### Similar Tolerance Levels to Water Deficit Between the Two Genotypes but Different Mechanisms Involved

The level of tolerance of each genotype to water deficit was estimated by measuring the overall plant growth at the end of the water deficit period (Figure 3). Because both genotypes were negatively affected to a similar extent, as illustrated by a similar decrease in the most integrative trait that is total plant biomass (Figure 3), we considered that Kayanne and Puget displayed the same level of tolerance to water deficit. This decrease in biomass acquisition induced by drought, classically observed in the literature, could be explained by a reduced carbon uptake arising from coordinated plant structural and functional changes, including a reduction of leaf area and a decrease in photosynthetic activity and RUE (Figure 5 and Table 1), although we cannot exclude a higher respiration or root exudation (not measured in the present study). Carbon partitioning within the plant was modified and the nodule compartment was the most severely plant organ affected by water deficit (Figure 6 and Table 2). This resulted in a decrease in SNF, a lowered NNI and nitrogen concentration (Figure 7 and Supplementary Figure S2). Altogether, these results are consistent with previous studies in pea (Lecoeur and Sinclair, 1996; Mahieu et al., 2009; Prudent et al., 2016) and other legume crops (Chaves et al., 2003, 2009). This decrease of SNF was not attributed to its functional component (nodule specific activity, sNFA Table 1), but rather to its structural component (nodule biomass, Figure 6C). Such strategy has already been observed in *Medicago truncatula*, when Jeudy et al. (2010) characterized structural rather than functional changes in SNF, in split root systems where a local suppression of SNF was applied through a partial root deprivation of N_2_ (Ar/O_2_ instead of air). This could illustrate the optimization of plant N supply at a lower carbon cost during a water deficit period.

Although the two genotypes displayed a similar level of tolerance toward water deficit on a plant biomass basis, our results showed that the mechanisms involved were distinct. One of the most significant differences between the two genotypes concerned the maintenance of water status during water deficit. This was revealed by a decrease in stomatal conductance for Kayanne over a longer period than Puget, together with a greater increase in water use efficiency (Figure 4). Moreover, Kayanne favored root growth at the expense of nodule growth while Puget only slightly decreased nodule growth while maintaining C allocation to the root system (Figure 6 and Table 2). The decrease in nodule biomass was explained by a reduction in individual nodule biomass, which was associated with a decrease in nodule number for Kayanne only (Figure 6D). This feature illustrates the genotype-dependent responses of nodules to water deficit in pea, and extends observations showing a concomitant decrease in the number and the size of the determinate nodules of soybean during water deficit (Sinclair et al., 1988; Fenta et al., 2012). To our knowledge, such legume intra-specific differences in adaptive responses to water deficit (relative to water status and C allocation to nodule compartment) have never been reported so far.

### Contrasted Strategies Between Genotypes During the Re-watering Period Might Explain Differences in Recovery Efficiency and Ultimately Resilience

**FIGURE 8:**
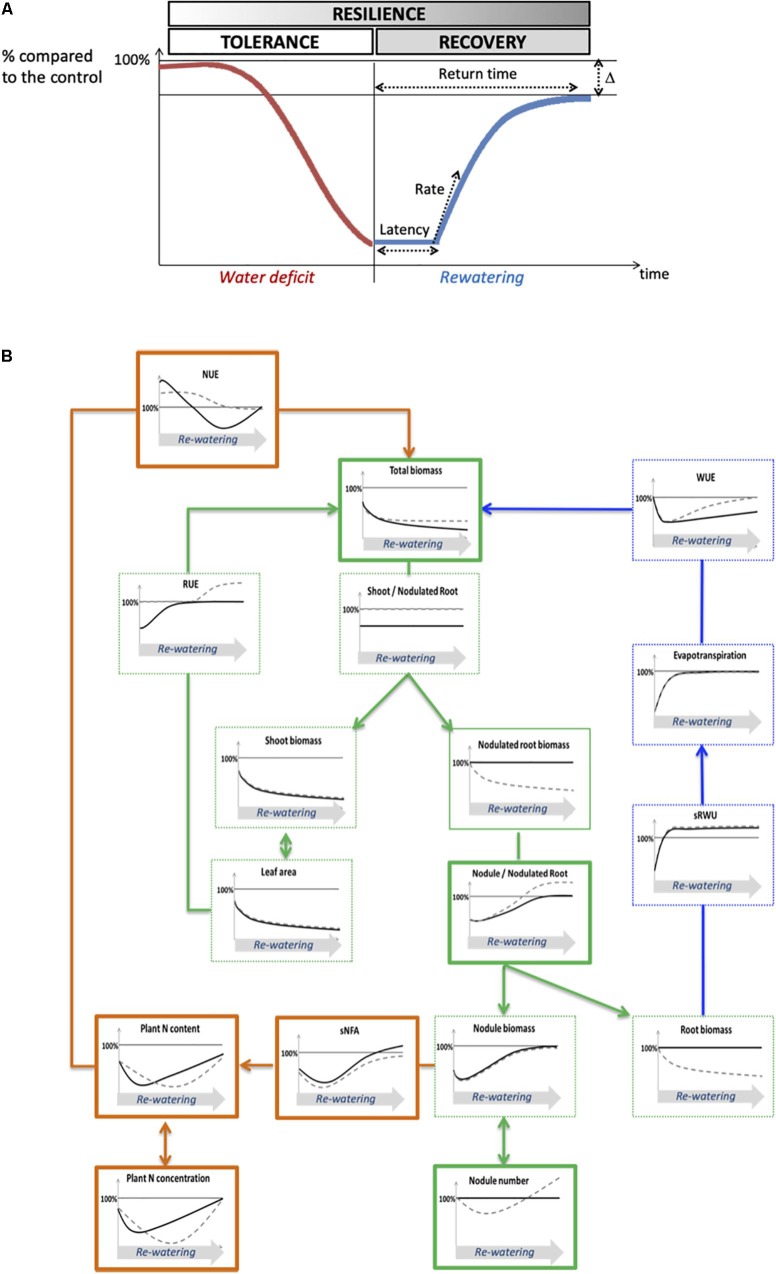
Theoretical and conceptual frameworks for the analysis of plant drought resilience. **(A)** Theoretical framework of plant resilience ability. The resilience process can be divided into drought tolerance and post-stress recovery. The curve represents the value of a given physiological process expressed as a percentage relative to the control plants, which decreases during water deficit, and recovers during the re-watering period until it reaches a plateau. The ability to recover can be characterized through four variables which are: the latency time to initiate a recovery, the rate of recovery, the return time to reach the plateau and delta (Δ), the difference of the value of the trait at the plateau between the well-watered plants and the plants subjected to water deficit. **(B)** Conceptual structure–function ecophysiological framework of plant recovery after water deficit. Variables related to carbon fluxes are in green, variables related to water fluxes are in blue, variables related to nitrogen fluxes are in orange. Kayanne genotype is in black and Puget genotype is in gray dotted line. Curves represent schematic data of plants having experienced a water deficit, and are expressed in percentage relative to the control plants. Variables shown in this study to play a major role in post-stress recovery in pea are framed with bold lines. NUE, nitrogen use efficiency; sNFA, specific nitrogen fixation activity; RUE, radiation use efficiency; WUE, water use efficiency; sRWU, specific root water uptake.

Although the two genotypes had similar reduced growth at the end of the water deficit, their yield were differentially affected at physiological maturity (Figure 2), suggesting that the resilience of each genotype could depend on the efficiency to recover during the re-watering period. The use of our conceptual structure–function framework linking C, N, and water fluxes at the whole plant level allowed us to compare the dynamics of recovery of each process with respect to the dynamics of plant biomass recovery, and to identify key processes which could explain a better recovery efficiency (Figure 8). To that aim, and for each trait, we considered various parameters such as the latency time to initiate the recovery, the rate of the process’s response and the gap (Δ) between the value of the process under WD and WW conditions (Figure 8A).

Based on plant total biomass, which is at the center of our conceptual framework (Figure 8B), there was no complete recovery after 2 weeks of re-watering. Nevertheless, a recovery response was initiated in both genotypes but with different latency times: after 3 to 7 days in Kayanne and after 10 to 15 days in Puget.

Because the latency time to initiate a recovery response for plant growth was not synchronized with the latency time to initiate a recovery for C-related traits (leaf area, photosynthesis, RUE), this suggests that other processes underlie overall plant recovery. Water fluxes, even if they were affected by re-watering, could neither explain differences between genotypes because they were not synchronized with the plant growth recovery response. A fast and complete recovery of evapotranspiration, associated with a lower WUE could reflect a delay between the reactivation of water uptake and the reactivation of water use for metabolism after re-watering, similarly to the delay observed between the reactivation of N uptake and N metabolism after a drought event in *Medicago truncatula* (Larrainzar et al., 2009).

The genotype-dependent dynamics of recovery of overall plant N status (NNI, Figure 7B; plant N concentration, Supplementary Figure S4) were very close to that of total biomass, suggesting that N acquisition could be a key process underlying plant recovery after water deficit in pea. This is consistent with the study from Lyon et al. (2016), which focused on metabolome and proteome responses of nodulated *Medicago truncatula* plants during drought and recovery and which suggested that the availability of an amino acids pool is essential for enabling a drought recovery, thus highlighting the tight link between N compound availability and biomass recovery. The relaunch of N acquisition through SNF could be explained by an increase in the intrinsic ability of the nodule to fix N_2_ (sNFA) or/and an increase of nodule biomass (which could result from increased nodule growth or increased nodule number). In our study, from a functional point of view, although sNFA was not significantly decreased at the end of the water deficit for both genotypes, it was lower after 7 days of re-watering for WD Puget plants when compared to WW plants but not for Kayanne. This can either mean that sNFA of WD Kayanne plants was maintained at a level similar to that of WW plants throughout the follow-up period, or that the sNFA decreased but rapidly recovered after 7 days of re-watering contrary to what was observed for Puget. This latter hypothesis is favored when looking at (i) the dynamics of plant nitrogen concentration and total nitrogen amount and (ii) because sNFA can recover rather quickly despite being sensitive to water deficit (Naya et al., 2007; Nasr Esfahani et al., 2014).

From a structural point of view, structural components of N_2_ fixation such as the nodule proportion in the nodulated root system fully recovered after re-watering, as previously reported for changes of plant N status (Jeudy et al., 2010; Prudent et al., 2016). Specifically, for Puget, a delay in the initiation of the second nodulation wave was observed, as well as an overcompensation for the nodule proportion in the nodulated root system explained by a higher number of nodules initiated during the second wave of nodulation for the WD plants (Figure 6D). We previously showed that in pea cv Caméor, the intensity of nodule initiation following drought was driven by the value of the NNI after drought (Prudent et al., 2016). Because N status in Puget was more impaired by drought and during a longer time than in Kayanne (NNI, Figure 7B), probably because this genotype favored C acquisition with a quick restoration of RUE together with a lesser C allocation to nodulated roots, it is tempting to speculate that Puget initiated an intensive nodule formation to offset its N deficiency. Over-compensatory recovery has already been reported by Xu et al. (2010) for mild water deficit in grass species, but only concerned traits related to C acquisition. Our study suggests that over-compensatory C allocation toward nodules during recovery in Puget was detrimental for the overall plant growth. Indeed, there is usually a trade-off for C use between roots and nodules to reach an optimal equilibrium between benefits related to SNF and C cost for nodule formation and functioning without impairing root development (Hacin et al., 1997; Tricot et al., 1997). In some cases, such as in hypernodulating mutants for which the autoregulation of the nodulation is disrupted (see the review from Mortier et al., 2012), many pleiotropic effects including shoot growth and yield depression have been reported (Novak et al., 2011). Our data suggest that a finely tuned nodule number initiation during the post-stress period is critical to ensure optimal N nutrition without excessive C costs (case of Kayanne), leading to plant growth recovery and contributing the N pool available for remobilization to the seeds later on during the reproductive period (Zeiher et al., 1982; Schiltz et al., 2005).

Thus, two different recovery strategies can be distinguished with (i) Kayanne which initiates its nitrogen nutrition recovery early, gradually leading to a complete growth recovery and (ii) Puget which initiates first its water status and RUE recoveries, then its nitrogen nutrition, in a later but faster manner, with some detrimental overcompensations with high carbon cost, finally leading to loss of yield. Altogether these results support our original working hypothesis, that a quick and strict adjustement of the number of nodules to plant growth needs is a key trait for an efficient post-drought recovery.

## Conclusion

The capacity of legume plants to be resilient when they face water deficit comprises their ability to tolerate the stress but also to efficiently recover post-stress. Our study highlights a genotype-dependent drought resilience of pea plants and contributes to the identification of key traits which could help in the design of pea ideotypes better adapted to fluctuating soil water conditions. Although the processes related to C, N and water fluxes did not display similar kinetics of recovery, we observed a synchronized recovery of plant growth and plant N nutrition. Our results emphasized for the first time that a quick recovery initiation of N acquisition, associated with a fine-tuning of nodule formation which allows benefits from SNF at low C cost, could be essential for yield stability after drought. As such, this study thus deepens our knowledge on post-drought recovery. It demonstrates that a strategy relying on maintaining its nitrogen status confers legume plant a better ability to post-drought recover than if the plant was addressing first its water and carbon status changes before its nitrogen status.

## Data Availability Statement

All datasets generated for this study are included in the article/Supplementary Material.

## Author Contributions

CS, MP, and VV conceived the project. CJ, CS, MC, MP, SG, and VV performed the experiments and contributed to the writing and approved the final manuscript. MC analyzed the data. CS, MC, MP, and VV wrote the publication.

## Conflict of Interest

The authors declare that the research was conducted in the absence of any commercial or financial relationships that could be construed as a potential conflict of interest.
