# Supplementary material for: Pea Efficiency of Post-drought Recovery Relies on the Strategy to Fine-Tune Nitrogen Nutrition
Source: Front Plant Sci. 2020 Feb 27;11:204. doi: 10.3389/fpls.2020.00204 (PMC7056749; doi:10.3389/fpls.2020.00204)
Supplement: Supplementary file 1 [file Data_Sheet_1.docx]

Supplementary Material

# Supplementary Figure S1:

**Evolution of the percentage of the water holding capacity of the substrate during the water deficit and subsequent re-watering.**

The water holding capacity of the substrate was estimated on 10 substrate aliquots by saturating the substrate with water and by drying it during 7 days at 110°C. Then, because each pot was weighted before each of the four daily waterings, the evolution of the water holding capacity of the substrate was calculated for each pot, four times a day.

# Supplementary Figure S2:

**Conceptual structure-function ecophysiological framework.**

NUE

Plant N content

Plant N concentration

sNFA

WUE

Evapotranspiration

sRWU

Shoot / Nodulated Root

Total Biomass

Shoot Biomass

RUE

Leaf Area

Nodulated Root Biomass

Nodule / Nodulated Root

Nodule Biomass

Root Biomass

Nodule Number

Conceptual structure-function ecophysiological framework. Variables related to carbon fluxes are in green, variables related to water fluxes are in blue, variables related to nitrogen fluxes are in orange.

NUE: Nitrogen Use Efficiency; sNFA: specific Nitrogen Fixation Activity; RUE: Radiation Use Efficiency; WUE: Water Use Efficiency; sRWU: soecific Root Water Uptake

# Supplementary Figure S3:

Pictures of shoots from genotypes Kayanne (A) and Puget (B) and nodulated roots from Kayanne (C, E) and Puget (D, F) after four-weeks growth.


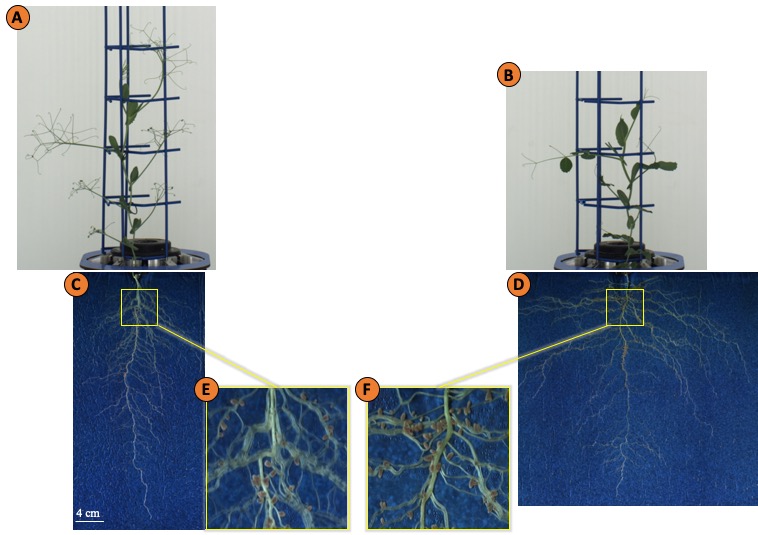


# Supplementary Figure S4:

**Effect of water deficit and subsequent re-watering on plant nitrogen related traits.**

**(A)** Plant nitrogen concentration was determined during the water deficit period (0, 7, 13 days) and after 3, 7 and 15 days of re-watering and **(B)** Nitrogen Use Efficiency (NUE) was calculated between two successive harvests. Kayanne is in black and Puget in grey. For each genotype, data are presented as a percentage relative to the control plants. Asterisks indicate Student’s t-test significant differences between control and water deficit plants for a given genotype (black asterisk for Kayanne, grey asterisk for Puget; p < 0.05, n=6).
